# Blue light triggered generation of reactive oxygen species from silica coated Gd_3_Al_5_O_12_:Ce^3+^ nanoparticles loaded with rose Bengal

**DOI:** 10.1016/j.dib.2018.08.072

**Published:** 2018-08-30

**Authors:** Akhil Jain, Rina Koyani, Carlos Muñoz, Prakhar Sengar, Oscar E. Contreras, Patricia Juárez, Gustavo A. Hirata

**Affiliations:** a Universidad Nacional Autónoma de México – Centro de Nanociencias y Nanotecnología, Km. 107 Carretera Tijuana-Ensenada, Ensenada, B.C. 22860, Mexico; b Posgrado en Nanociencias, Centro de Investigación Científica y de Educación Superior de Ensenada, Carretera Ensenada-Tijuana No. 3918, Zona Playitas, C.P. 22860 Ensenada, B.C., Mexico; c Departamento de Innovación Biomédica, Centro de Investigación Científica y de Educación Superior de Ensenada, Carretera Ensenada-Tijuana No. 3918, Zona Playitas, C.P. 22860 Ensenada, B.C., Mexico

## Abstract

This data article provide results of the studies conducted to develop a mesoporous silica coated Gd_2.98_Ce_0.02_Al_5_O_12_ nanoparticles loaded with a photosensitizer dye rose Bengal (RB) system (GAG@mSiO_2_@RB) capable of producing reactive oxygen species (ROS) upon exposure to blue light. The data reported here is related with Jain et al. (2018) [1]. It contains histogram of particle size distribution, cathodoluminescence (CL), photoluminescence spectra and there spectral overlap with the absorption spectra of RB, a graph showing the loading percentage of RB at different concentrations. Moreover, the data indicating ROS generation evaluated using 1,2-diphenylisobenzofuran (DPBF) assay and the viability of MDA-MB-231 cells upon exposure with different concentration of GAG@mSiO_2_ nanoparticles, upon exposure with blue light is also included in the data.

## Specifications table

**Table:** 

Subject area	*Nanotechnology*
More specific subject area	*Material Science and Nanomedicine*
Type of data	*Graphs and Figures*
How data was acquired	• *CL spectra was acquired from measurements in a Gatan mono-CL system in UV–Vis range coupled with scanning electron microscope (JSM-7800F, JEOL).* • *RB loading percentage was determined using UV–vis Cary 60 spectrophotometer.* • *PL spectra were obtained using a fluorescent spectrophotometer (Hitachi F-7000) equipped with a 150 W Xenon lamp.*
Data format	*Filtered*
Experimental factors	• *The as-synthesized nanoparticles were annealed at 1100 °C for 3 h before any further usage.* • *The samples for TEM analysis were prepared by 15 min ultrasonic dispersion of nanoparticles in isopropanol. Histogram of particle size distribution was acquired from diameter of 80 different images of nanoparticles obtained from TEM.*
Experimental features	*The sol–gel synthesized* GAG *nanoparticles were coated with mesoporous silica and later, RB was loaded inside these mesopores. Finally, ROS generation upon exposure with blue light was confirmed by DPBF assay.*
Data source location	*Ensenada, Baja California, Mexico.*
Data accessibility	*Data is available with this article.*
Related research article	*The data presented in this article is related to the research article: Akhil Jain, Rina Koyani, Carlos Muñoz, Prakhar Sengar, Oscar E. Contreras, Patricia Juárez, Gustavo A. Hirata, Magnetic-Luminescent Cerium-Doped Gadolinium Aluminum Garnet Nanoparticles for Simultaneous Imaging and Photodynamic Therapy of Cancer Cells, Journal of Colloids and Interface Science, In press.*

## Value of the data

• The data provide useful evidences on luminescent properties of the GAG nanoparticles upon exposure with high energy electrons as well as visible photons. These properties of the proposed gadolinium containing nanoparticles together with negligible toxicity *in vitro*, could allow their future application for *in vivo* multimodal imaging.
• The data demonstrates that the studied nanocomposite upon exposure to high energy electrons emit visible photons that can be readily absorbed by RB to generate ROS. Thus providing an important evidence that could serve as platform for future research focused towards development of novel strategies for photodynamic therapy (PDT) of deep tumors.
• The data could encourage future research directed towards magnetically guided deep PDT.

## 1 Data

This data article contains information related to the research article entitled “Magnetic-Luminescent GAG:Ce^3+^ Nanoparticles for Simultaneous Imaging and Photodynamic Therapy of Cancer Cells” in Journal of Colloid and Interface Science [1]. In this article, we report the ability of mesoporous silica coated GAG nanoparticles loaded with RB to generate ROS upon exposure with blue light (*λ*_ex_ = 470 nm).

## 2 Experimental design, materials, and methods

**Fig. 1:**
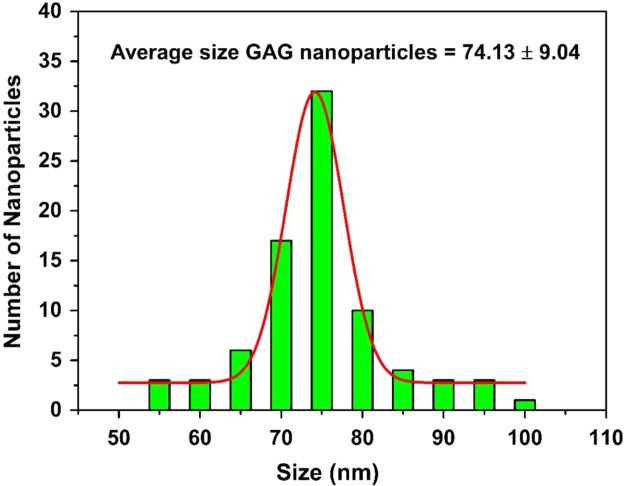
Histogram of size distribution of GAG nanoparticles synthesized by sol–gel method using TEM data.

**Fig. 2:**
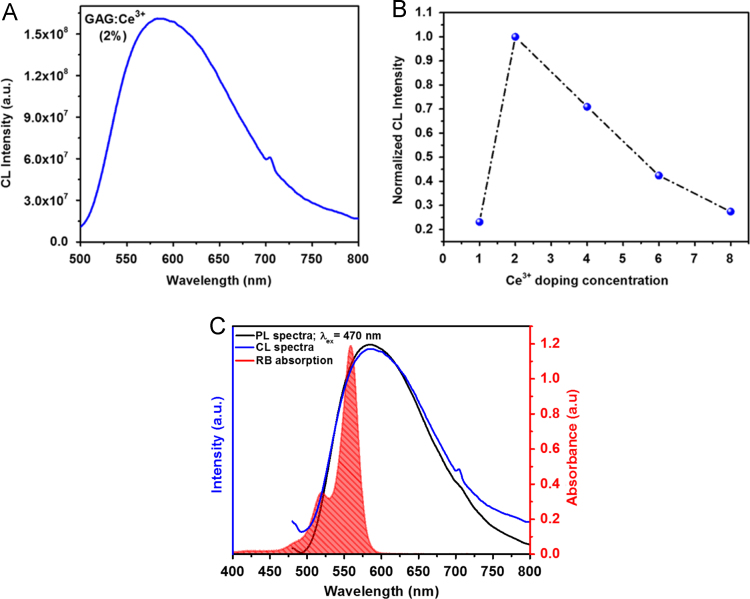
Cathodoluminescence (CL) spectra of GAG nanoparticles upon excitation with high energy electrons (15 keV). (A) GAG:Ce^3+^ (2%), (B) Normalized CL intensity of GAG nanoparticles doped with different cerium concentration, and (C) Spectral overlap between CL and PL spectra of GAG nanoparticles with absorption of RB.

**Fig. 3:**
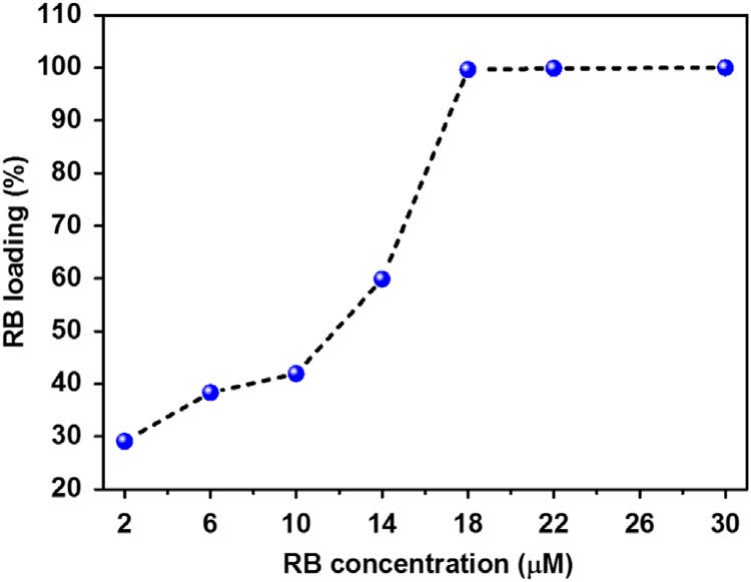
Loading percentage of RB at different concentrations in to GAG@mSiO_2_ nanoparticles.

**Fig. 4:**
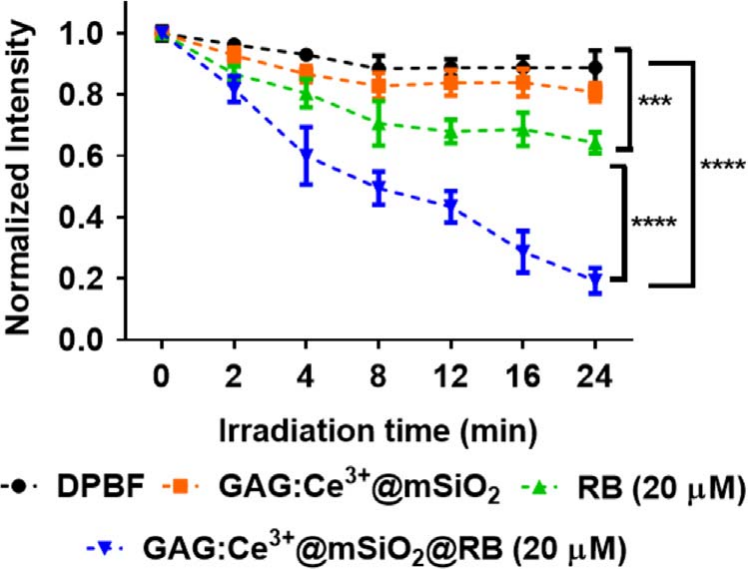
Measurement of ROS generation. Singlet oxygen species generation upon excitation with blue light (470 nm, 20 mW/cm^2^) measured using DPBF assay. Experiment was performed using triplicates and the data is expressed as mean ± S.E.M. Statistical significance at ****p < 0.0001 was calculated using 2-way ANOVA with Tukey post-test.

**Fig. 5:**
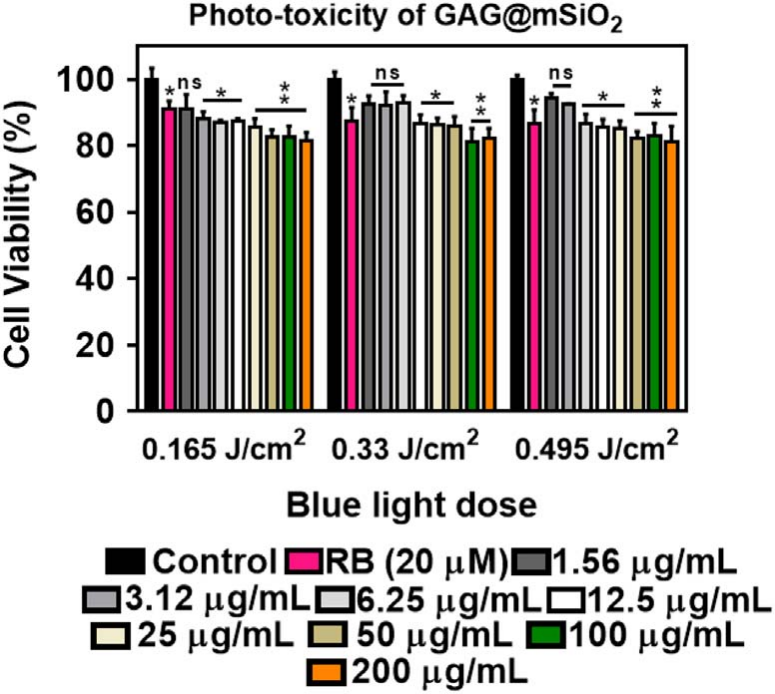
Photo-toxicity of GAG@mSiO_2_ nanoparticles. Cell viability of MDA-MB-231 cells calculated using MTT assay upon exposure with blue light at different doses of 0.165, 0.33 and 0.495 J/cm^2^. Experiment was performed using quadruplicates and the data are expressed mean ± S.E.M. Statistical significance at **p < 0.01 was calculated using 2-way ANOVA with Tukey post-test.

First, we showed the particle size distribution of GAG nanoparticle that were originally synthesized by sol–gel method (Fig. 1). Next, we demonstrated that upon excitation with high energy electrons (15 keV) and blue light (*λ*_ex_ = 470 nm) the GAG nanoparticles shows a broad emission peak ranging from 510 to 750 nm with maximum intensity at 585 nm (Fig. 2A). We showed that doping with 2% atomic concentration of cerium inside GAG host lattice produces highest emission intensity (Fig. 2B). The emission spectra (cathodoluminescence and PL emission) of GAG nanoparticles was analyzed to determine the spectral overlap with the absorption of a photosensitizer dye RB (Fig. 2C). The GAG nanoparticles were coated with mesoporous silica and then loaded with RB to yield GAG@mSiO_2_@RB nanocomposite. The loading percentage of RB inside mesoporous silica coated GAG nanoparticles was determined by UV–vis spectroscopy (Fig. 3). Later, the ROS producing ability of GAG@mSiO_2_@RB nanocomposite upon exposure with blue light (470 nm, 20 mW/cm^2^) was analyzed by DPBF assay and compared with free RB and GAG@mSiO_2_ controls (Fig. 4). Finally, we analyzed the cellular toxicity of GAG@mSiO_2_ nanoparticles in presence of blue light (*λ*_ex_ = 470 nm) at different doses using MTT analysis (Fig. 5).

### 2.1 Nanoparticle synthesis and mesoporous silica coating

The nanoparticles were synthesized using sol–gel method as reported in [2]. Surface of GAG nanoparticles was coated with mesoporous silica using the protocol used in [1]. The Photoluminescence excitation and emission spectra were obtained using a fluorescent spectrophotometer (Hitachi F-7000) equipped with a 150 W Xenon lamp. Cathodoluminescence spectra of the synthesized GAG samples was acquired using a Gatan mono-CL system in UV–Vis range coupled with scanning electron microscope (JSM-7800F, JEOL). UV–vis and photoluminescence spectra were recorded using a 10 mm rectangular quartz cell (Starna Cells Inc.) and repeated for at least three times.

### 2.2 RB loading

GAG@mSiO_2_ nanoparticles (10 mg/mL) ultrasonically dispersed in water were added with different concentrations of RB ranging from 1 to 40 µM. The reaction was continued under constant rotation for another 12 h under dark. Finally, the obtained GAG@mSiO_2_@RB nanocomposite were washed several times with water until the supernatant was colorless, to remove any excess RB. The loading percentage of RB inside the GAG@mSiO_2_ nanoparticles was determined by UV–vis absorption spectroscopy.

### 2.3 Measurement of ROS generation

Generation of singlet oxygen was detected using 1,2-diphenylisobenzofuran (DPBF) as reported in [3]. In a typical protocol DPBF (2 mM) was dissolved in DMSO and mixed with 2 mg GAG@mSiO_2_@RB (RB loading concentration = 20 µM) ultrasonically dispersed in water. The mixture was irradiated with blue light (470 nm, 20 mW/cm^2^) for different time intervals (ranging from 0 to 25 min). ROS generation was confirmed by analyzing the photoluminescence emission spectra (*λ*_em_ = 485 nm) of DPBF upon excitation with a wavelength of 410 nm. All the measurement were performed in triplicates.

### 2.4 MTT assay

MDA-MB-231 cells were seeded at a density of 10^4^ cells per well and incubated for 24 h. Cells were then treated with different concentration (1.56–200 µg/mL) of GAG@mSiO_2_ nanoparticles and incubated for another 24 h. Later, the cells were exposed to blue light (20 mW/cm^2^ and *λ* = 470 nm) at different doses of 0.165 J/cm^2^ (15 min), 0.33 J/cm^2^ (30 min) and 0.495 J/cm^2^ (45 min). After 24 h, 20 µL of MTT solution was added and incubated for 5 h. Finally, 100 µL of a stop buffer (0.01 M HCl containing 10% SDS) was added, and the plates were incubated for 20 h. The absorbance of the plate at 570 nm was measured using an Epoch microplate reader (Biotek).
